# Lymph node metastasis in patients with hepatocellular carcinoma using machine learning: a population-based study

**DOI:** 10.3389/fonc.2025.1601985

**Published:** 2025-07-11

**Authors:** Li Yuqin, Li Hongyan, Li Hongyuan, Li Tingting, He Kun, Fang Jie, Han Yunhui

**Affiliations:** ^1^ Department of Obstetrics and Gynecology, Jinan Central Hospital, Jinan, China; ^2^ School of Clinical Medicine, Southwest Medical University, Luzhou, Sichuan, China; ^3^ Department of Anesthesiology, The Affiliated Traditional Chinese Medicine Hospital, Southwest Medical University, Luzhou, China; ^4^ Luzhou Key Laboratory of Research for Integrative on Pain and Perioperative Organ Protection, Luzhou, China; ^5^ Department of Health Management Center, Sichuan Clinical Research Center for Cancer, Sichuan Cancer Hospital & Institute, Sichuan Cancer Center, University of Electronic Science and Technology of China, Chengdu, China; ^6^ Clinical Research Institute, The Affiliated Hospital, Southwest Medical University, Luzhou, China; ^7^ Department of Respiratory Medicine, Dezhou People’s Hospital, Dezhou, Shandong, China

**Keywords:** hepatocellular carcinoma, machine learning, predictive model, lymph node metastasis, logistic regression

## Abstract

**Aim:**

This study aims to develo\p a population-adapted machine learning-based prediction model for hepatocellular carcinoma (HCC) lymph node metastasis (LNM) to identify high-risk patients requiring intensive surveillance.

**Methods:**

Data from 23511 HCC patients in the SEER database and 57 patients from our hospital were analyzed. Seven LNM risk indicators were selected. Four machine learning algorithms—decision tree (DT), logistic Regression (LR), multilayer perceptron (MLP), and extreme gradient boosting (XGBoost)—were employed to construct prediction models. Model performance was evaluated using area under the curve, accuracy, sensitivity, and specificity.

**Results:**

Among 23511 SEER patients, 1679 (7.14%) exhibited LNM. Race, Sequence number, Tumor size, T stage and AFP were identified as independent predictors of LNM. The LR model achieved optimal performance (area under the curve: 0.751; accuracy: 0.707; sensitivity: 0.711; specificity: 0.661). External validation with 57 patients from our hospital confirmed robust generalizability (area under the curve: 0.73; accuracy: 0.737; sensitivity: 0.829; specificity: 0.5), outperforming other models.

**Conclusions:**

The LR-based model demonstrates superior predictive capability for LNM in HCC, offering clinicians a valuable tool to guide personalized therapeutic strategies.

## Background

Hepatocellular carcinoma (HCC) ranks as the sixth most common cancer worldwide and is the most prevalent—and deadliest—form of primary liver cancer, representing the third leading cause of cancer-related mortality (1). Its etiological risk factors exhibit marked geographic heterogeneity, with strong associations to hepatitis B virus (HBV) and hepatitis C virus (HCV) infections, alcoholic liver disease, and metabolic syndrome (2). Despite advances in therapeutic modalities—including surgical resection, liver transplantation, and local ablation (3)—long-term outcomes for HCC patients remain dismal, with a 5-year survival rate below 20% (4). The often insidious onset of HCC frequently delays diagnosis until advanced stages, increasing the likelihood of lymph node metastasis (LNM) (5), a pivotal event in HCC progression that significantly worsens prognosis (6). Patients with LNM have a median survival of only 5.8 months, compared to 16.3 months for those without nodal involvement (7), and nodal metastases preclude curative resection while indicating systemic disease dissemination (8).

Early, accurate prediction of LNM is therefore essential for individualized treatment planning and prognostic stratification. Current clinical assessment relies primarily on imaging modalities—such as computed tomography (CT) and magnetic resonance imaging (MRI)—and histopathological evaluation. Proposed imaging predictors, including hilar invasion or a short-axis lymph node diameter ≥9 mm, have demonstrated variable sensitivity and specificity, reflecting the low incidence of HCC nodal metastasis (1.23%–7.5%) and cohort heterogeneity (9–11). Traditional prediction tools, such as TNM staging–based nomograms, typically incorporate only single clinical variables and neglect tumor biology and multidimensional patient data; retrospective study designs further introduce selection bias, and the lack of external validation limits generalizability (9). For example, an HBV-focused LNM prediction model experienced a drop in area under the receiver operating characteristic curve (AUC) to 0.68 upon external validation, underscoring its restricted applicability (12).

Machine learning (ML) offers a promising alternative by integrating heterogeneous, multimodal data (e.g., radiomics, genomics, clinical variables) through nonlinear algorithms to reveal latent predictive patterns (13). In other malignancies, ML-based models have outperformed conventional approaches—for instance, an artificial neural network predicting LNM in early-stage colorectal cancer achieved an AUC of 0.859 (14), and ML integration of clinical data improved thyroid cancer diagnostic accuracy (15). However, ML-based prediction of LNM in HCC remains scarce, with existing studies limited to small, single-center cohorts lacking population-level validation.

The Surveillance, Epidemiology, and End Results (SEER) program of the U.S. National Cancer Institute provides a large, multicenter, patient-centered database encompassing demographic, tumor, pathological, and follow-up information. Leveraging multidimensional SEER data alongside a substantial HCC cohort from our hospital, this study aims to develop and externally validate a population-adapted ML model for predicting LNM in HCC, thereby facilitating early identification of high-risk patients who may benefit from intensified surveillance and tailored therapeutic strategies.

## Materials and methods

### Patient information

Data were obtained from the SEER database, a globally recognized cancer registry. LN status was determined according to the 7th edition of the American Joint Committee on Cancer (AJCC) Tumor-Node-Metastasis (TNM) staging system, using both imaging and pathological evidence.

Inclusion criteria comprised histologically confirmed HCC patients diagnosed between 2010 and 2015, age ≥ 20 years, complete clinical and treatment records, and documented LNM status. Exclusion criteria were non-HCC liver malignancies, incomplete data, or unknown LN status. After screening, 23511 patients were enrolled and randomly split into a training cohort (n=16459) and an internal test cohort (n=7052) at a 7:3 ratio.

**Figure 1:**
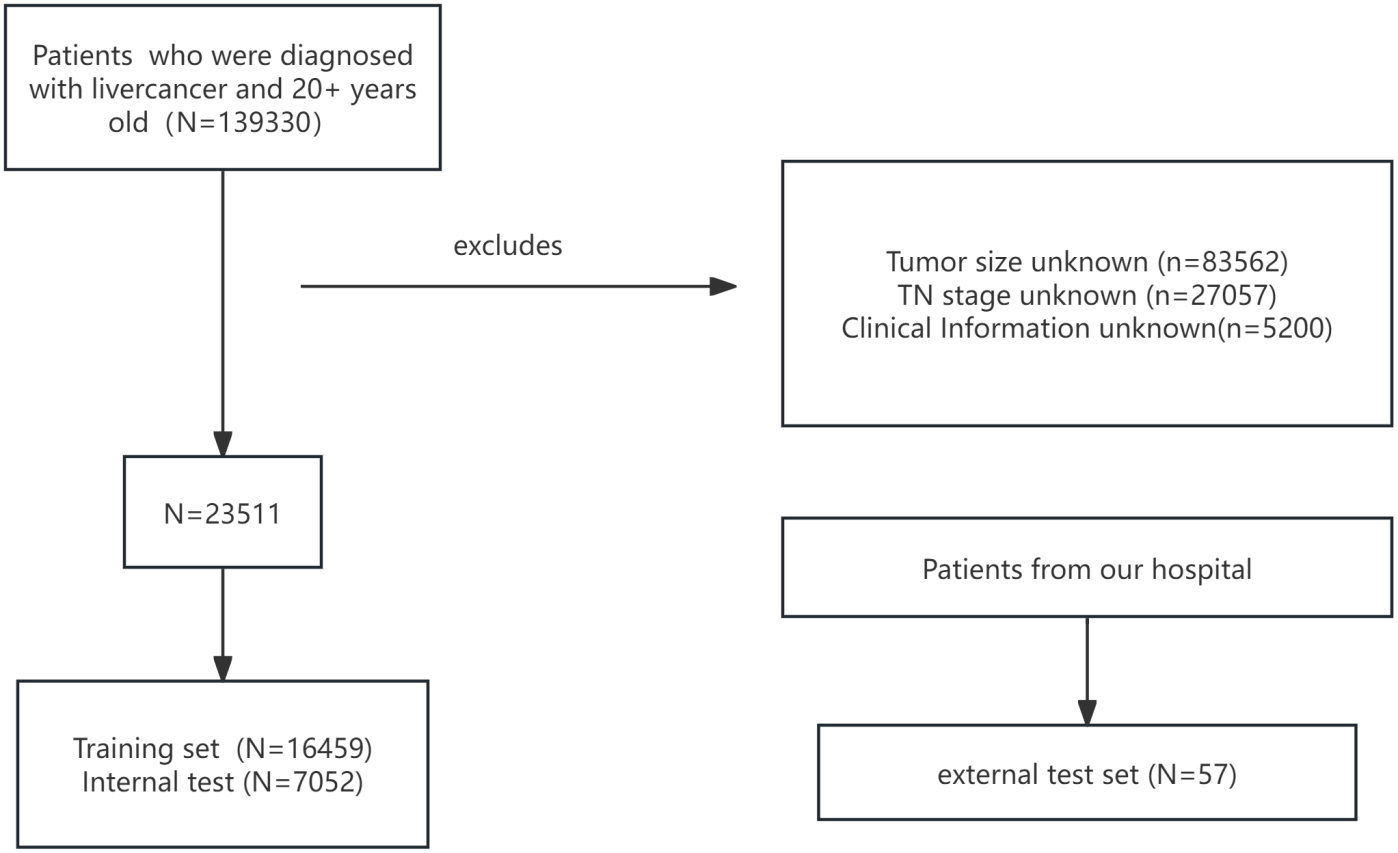
Patient selection flowchart.

An external validation cohort of 57 HCC patients from our hospital was included to assess model generalizability. The patient selection workflow is shown in **Figure 1**. Data extraction and verification were performed independently by three investigators. The study received ethics committee approval, and all patients provided informed consent; analyses were conducted anonymously to ensure confidentiality.

### Data preprocessing and feature selection

Key variables—including age, sex, race, AJCC TN stage, tumor size, sequence number, and alpha-fetoprotein (AFP)—were extracted using SEER*Stat (v8.4.4) and reviewed by clinicians. Age was dichotomized (<60 vs ≥60 years). In the training cohort, univariate logistic regression (LR) identified predictors of LN metastasis (p<0.05). Stepwise regression (forward selection, backward elimination, and bidirectional selection) estimated odds ratios (ORs) with 95% confidence intervals (CIs). All analyses were performed in R (v4.4.2).

### Model development and performance evaluation

Four ML algorithms—decision tree (DT), logistic regression (LR), multilayer perceptron (MLP), and extreme gradient boosting (XGBoost)—were used to build prediction models. To address class imbalance, a 1:1 ratio undersampling technique was incorporated into the preprocessing pipeline to ensure a balanced distribution of the target variable. Model performance was evaluated by area under the receiver operating characteristic curve (AUC), accuracy, sensitivity, and specificity. Variable importance plots were generated for all four models, followed by external validation.Additionally, pairwise comparisons of AUC differences were conducted using the DeLong test. The best-performing model was then selected, and a visualized clinical risk prediction nomogram was constructed based on this model.

### Correlation analysis and variable importance

Following feature selection, Spearman correlation analysis quantified inter-variable associations, classified as low (0–0.4), moderate (0.4–0.7), or high (≥0.7). For each model, variable importance was ranked using a permutation-based method.

## Results

### Patient characteristics

After screening, 23511 patients were included. All eight variables showed no significant differences between the training and internal test. In the external test, the variable race lacked a p-value due to the presence of only Asian individuals. (**Table 1**). **Table 2** demonstrates that all features differed significantly across datasets (p < 0.05).

**Table 1:** Characteristics in the training, internal test, and external test cohorts.

Variables	Training set (N=16459)	Internal test (N=7052)	p.overall	External test (N=57)	p.overall
Age:			0.909		0.099
<60	5951 (36.2%)	2556 (36.2%)		33 (57.9%)	
>=60	10508 (63.8%)	4496 (63.8%)		24 (42.1%)	
Sex:			0.914		0.66
Female	3819 (23.2%)	1631 (23.1%)		7 (12.3%)	
Male	12640 (76.8%)	5421 (76.9%)		50 (87.7%)	
Race:			0.889		.
Asian	2781 (16.9%)	1187 (16.8%)		57 (100%)	
American Indian	234 (1.42%)	104 (1.47%)		0 (0%)	
Black	2095 (12.7%)	874 (12.4%)		0 (0%)	
White	11349 (69.0%)	4887 (69.3%)		0 (0%)	
Sequence number:			0.143		0.483
One primary only	13519 (82.1%)	5735 (81.3%)		44 (77.2%)	
More than one primary	2940 (17.9%)	1317 (18.7%)		13 (22.8%)	
Tumorsize:			0.177		0.67
D < 3cm	5122 (31.1%)	2099 (29.8%)		27 (47.4%)	
3cm ≤ D < 5cm	4312 (26.2%)	1854 (26.3%)		15 (26.3%)	
5cm ≤ D < 10cm	4510 (27.4%)	2001 (28.4%)		12 (21.1%)	
D ≥ 10cm	2515 (15.3%)	1098 (15.6%)		3 (5.26%)	
T stage:			0.38		0.67
T1	7552 (45.9%)	3193 (45.3%)		27 (47.4%)	
T2	4152 (25.2%)	1842 (26.1%)		15 (26.3%)	
T3	4201 (25.5%)	1799 (25.5%)		12 (21.1%)	
T4	554 (3.37%)	218 (3.09%)		3 (5.26%)	
AFP:			0.587		0.584
Negative	4713 (28.6%)	1994 (28.3%)		20 (35.1%)	
Positive	11746 (71.4%)	5058 (71.7%)		37 (64.9%)	
N:			0.995		1
N0	15283 (92.9%)	6549 (92.9%)		41 (71.9%)	
N1	1176 (7.15%)	503 (7.13%)		16 (28.1%)	

T stage, tumor stage; AFP, alpha-fetoprotein; N stage, node stage.

**Table 2:** Characteristics of the patients presenting with and without lymph node metastases.

Variables	[ALL] (N=*23511)*	N0 (N=*21832*)	N1 (N=*1679*)	p.overall
Age:				0.013
<60	8507 (36.2%)	7852 (36.0%)	655 (39.0%)	
>=60	15004 (63.8%)	13980 (64.0%)	1024 (61.0%)	
Sex:				<0.001
Female	5450 (23.2%)	5142 (23.6%)	308 (18.3%)	
Male	18061 (76.8%)	16690 (76.4%)	1371 (81.7%)	
Race:				<0.001
Asian or Pacific Islander	3968 (16.9%)	3747 (17.2%)	221 (13.2%)	
American Indian	338 (1.44%)	306 (1.40%)	32 (1.91%)	
Black	2969 (12.6%)	2714 (12.4%)	255 (15.2%)	
White	16236 (69.1%)	15065 (69.0%)	1171 (69.7%)	
Sequence number:				<0.001
One primary only	19254 (81.9%)	17788 (81.5%)	1466 (87.3%)	
More than one primary	4257 (18.1%)	4044 (18.5%)	213 (12.7%)	
Tumorsize:				<0.001
D < 3cm	7221 (30.7%)	7052 (32.3%)	169 (10.1%)	
3cm ≤ D < 5cm	6166 (26.2%)	5887 (27.0%)	279 (16.6%)	
5cm ≤ D < 10cm	6511 (27.7%)	5823 (26.7%)	688 (41.0%)	
D ≥ 10cm	3613 (15.4%)	3070 (14.1%)	543 (32.3%)	
T stage:				<0.001
T1	10745 (45.7%)	10458 (47.9%)	287 (17.1%)	
T2	5994 (25.5%)	5712 (26.2%)	282 (16.8%)	
T3	6000 (25.5%)	5076 (23.3%)	924 (55.0%)	
T4	772 (3.28%)	586 (2.68%)	186 (11.1%)	
AFP:				<0.001
Negative	6707 (28.5%)	6427 (29.4%)	280 (16.7%)	
Positive	16804 (71.5%)	15405 (70.6%)	1399 (83.3%)	

T stage, tumor stage; AFP, alpha-fetoprotein; N stage, node stage.

### Univariate and multivariate logistic regression analyses

Univariate logistic regression identified seven factors significantly associated with lymph node metastasis (LNM; p < 0.05): age, sex, race, sequence number, tumor size, T stage and AFP status (**Table 3**). In multivariate analysis, the presence of multiple primary tumors was an independent protective factor against LNM. Independent risk factors included increasing age (≥60 years), male sex, American Indian, Black, and White race, larger tumor size (3cm ≤ D < 5cm, 5cm ≤ D < 10cm, D ≥ 10cm), advanced T stage (T2, T3, T4), and positive AFP levels.

**Table 3:** Univariable and multivariable logistic regression analyses of risk factors for lymph node metastasis.

Variables	Univariable		Multivariable	
	OR	Value of P	OR	Value of p
Age:				
< 60	Reference	Reference	Reference	Reference
≥ 60	0.87	0.028		
Sex:				
Female	Reference	Reference	Reference	Reference
Male	1.38	<0.001	1.13	0.132
Race:				
Asian or Pacific Islander	Reference	Reference	Reference	Reference
American Indian	1.96	0.003	2.23	0.001
Black	1.47	0.001	1.38	0.006
White	1.27	0.007	1.5	<0.001
Sequence number:				
One primary only	Reference	Reference	Reference	Reference
More than one primary	0.64	<0.001	0.79	0.014
Tumor size:				
D < 3cm	Reference	Reference	Reference	Reference
3cm ≤ D < 5cm	1.88	<0.001	1.61	0.001
5cm ≤ D < 10cm	4.62	<0.001	2.24	<0.001
D ≥ 10cm	7.34	<0.001	3.16	<0.001
T stage:				
T1	Reference	Reference	Reference	Reference
T2	1.83	<0.001	1.98	<0.001
T3	6.78	<0.001	4.17	<0.001
T4	11.33	<0.001	7.1	<0.001
AFP:				
Negative	Reference	Reference	Reference	Reference
Positive	2.2	<0.001	1.48	<0.001

Univariable, univariable regression; Multivariable, multivariable regression; OR, odds ratio.

### Correlation analysis

**Figure 2:**
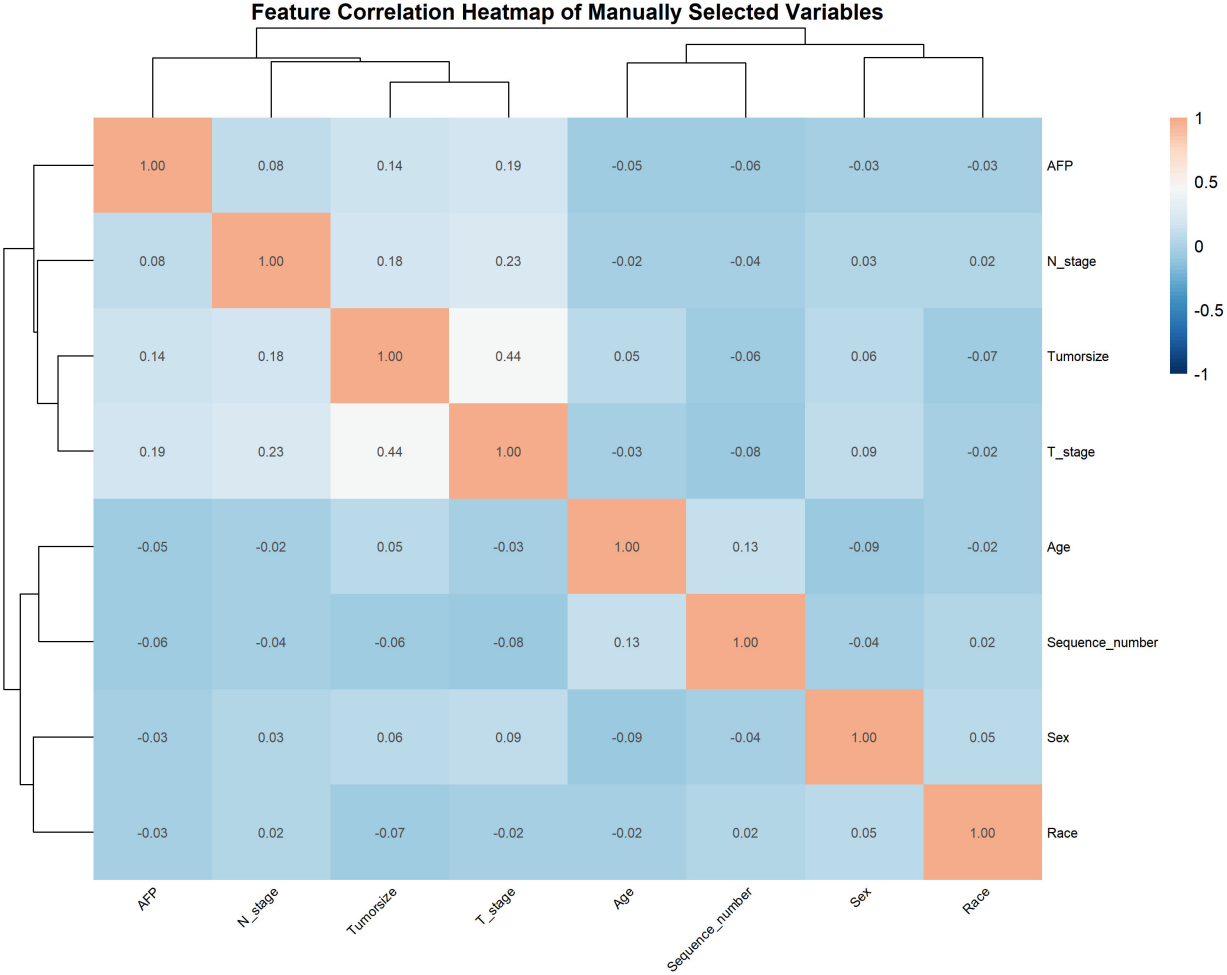
Heatmap of the correlation of patients’ clinical and pathological features.

Spearman’s rank correlation coefficients among the seven predictors were visualized via heatmap (**Figure 2**). T stage and tumor size showed a moderate positive correlation (p = 0.44), reflecting their joint association with tumor progression.

### Performance of machine learning algorithms

**Figure 3:**
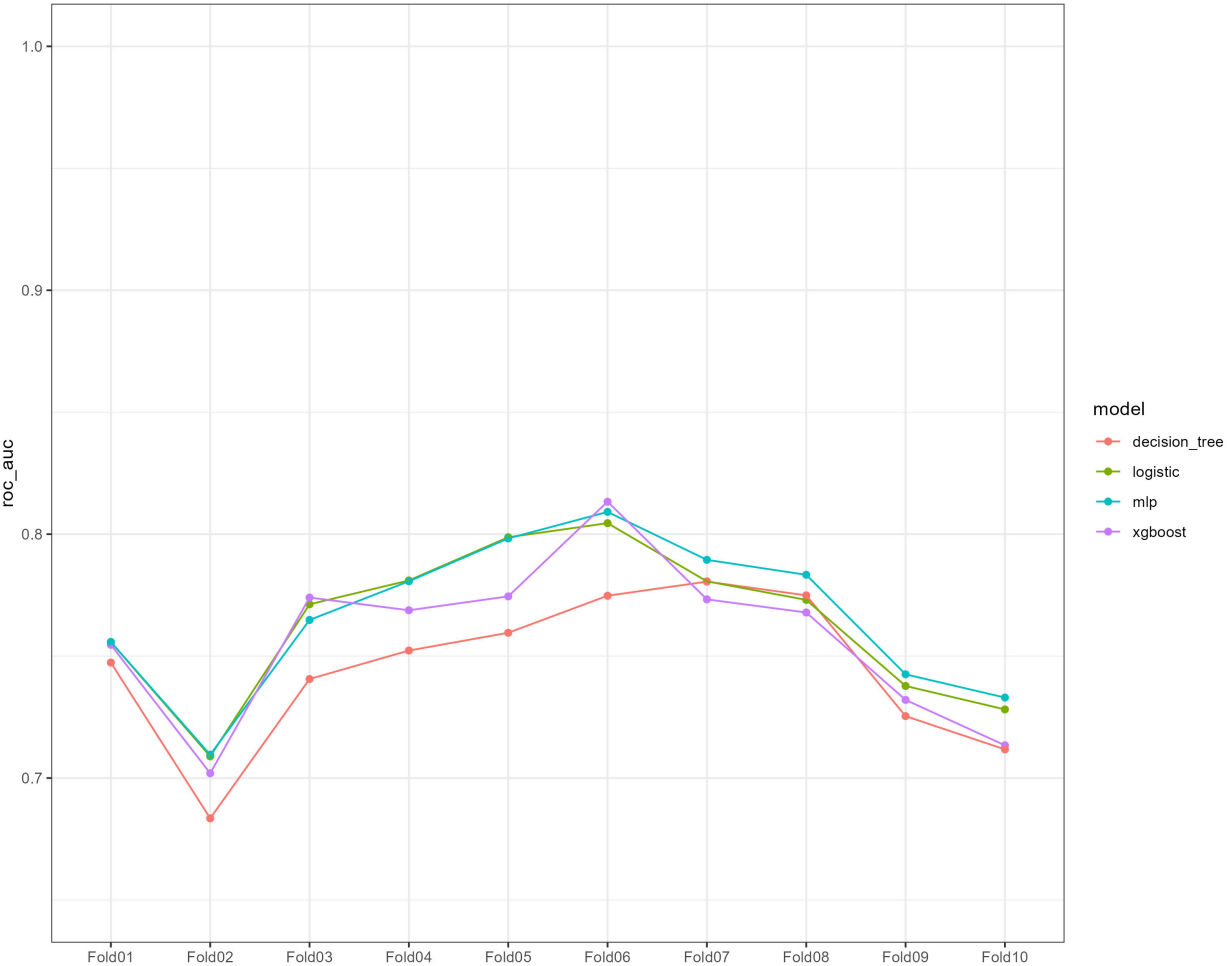
Ten-fold cross-validation of the receiver operating characteristic curves of the four machine learning models in the training cohort.

**Figure 4:**
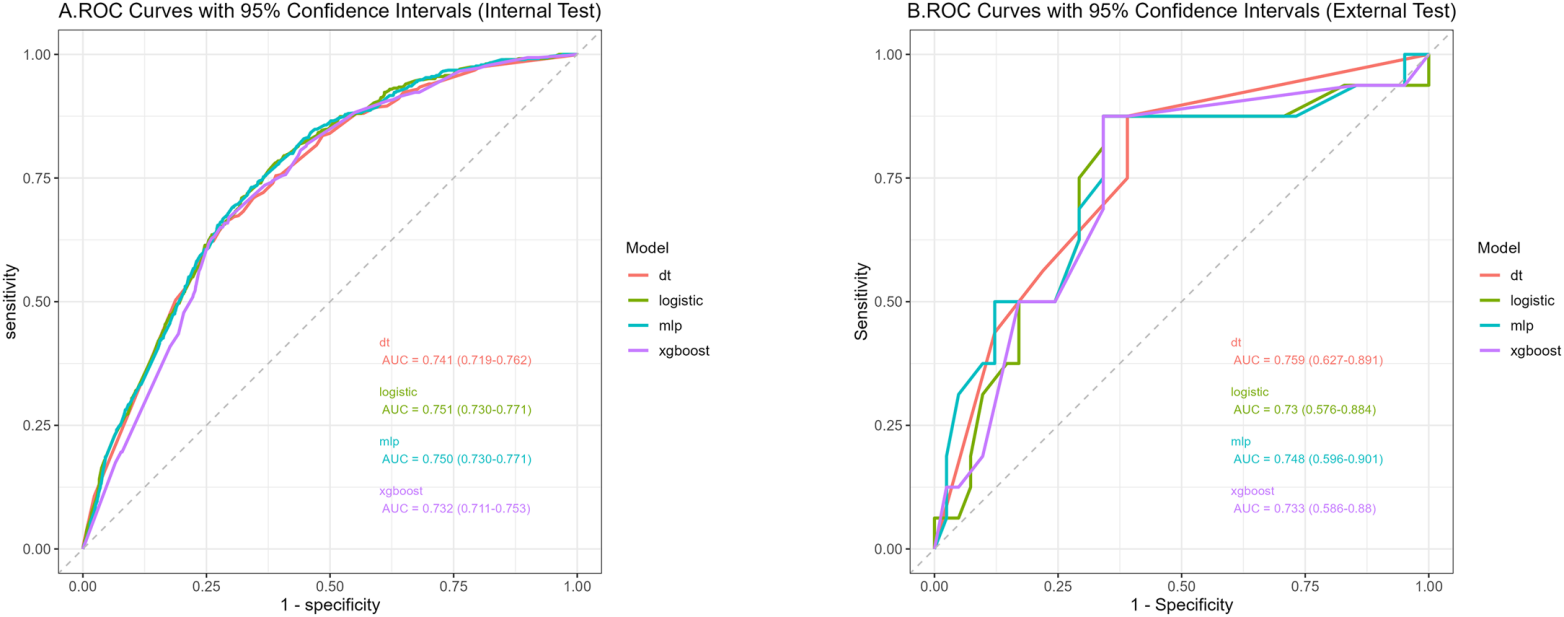
Receiver operating characteristic curves of the four algorithms in the internal **(A)** and external tests **(B)**.

**Figure 5:**
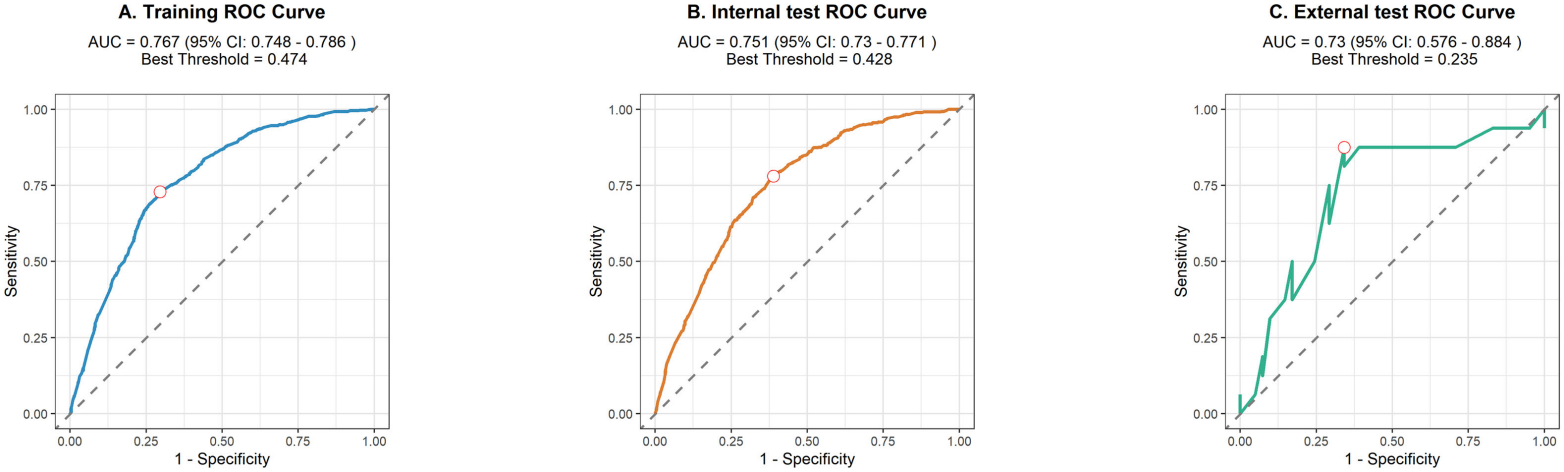
The ROC of LR on the training set **(A)**, internal test set **(B)**, and external test set **(C)**.

**Figure 6:**
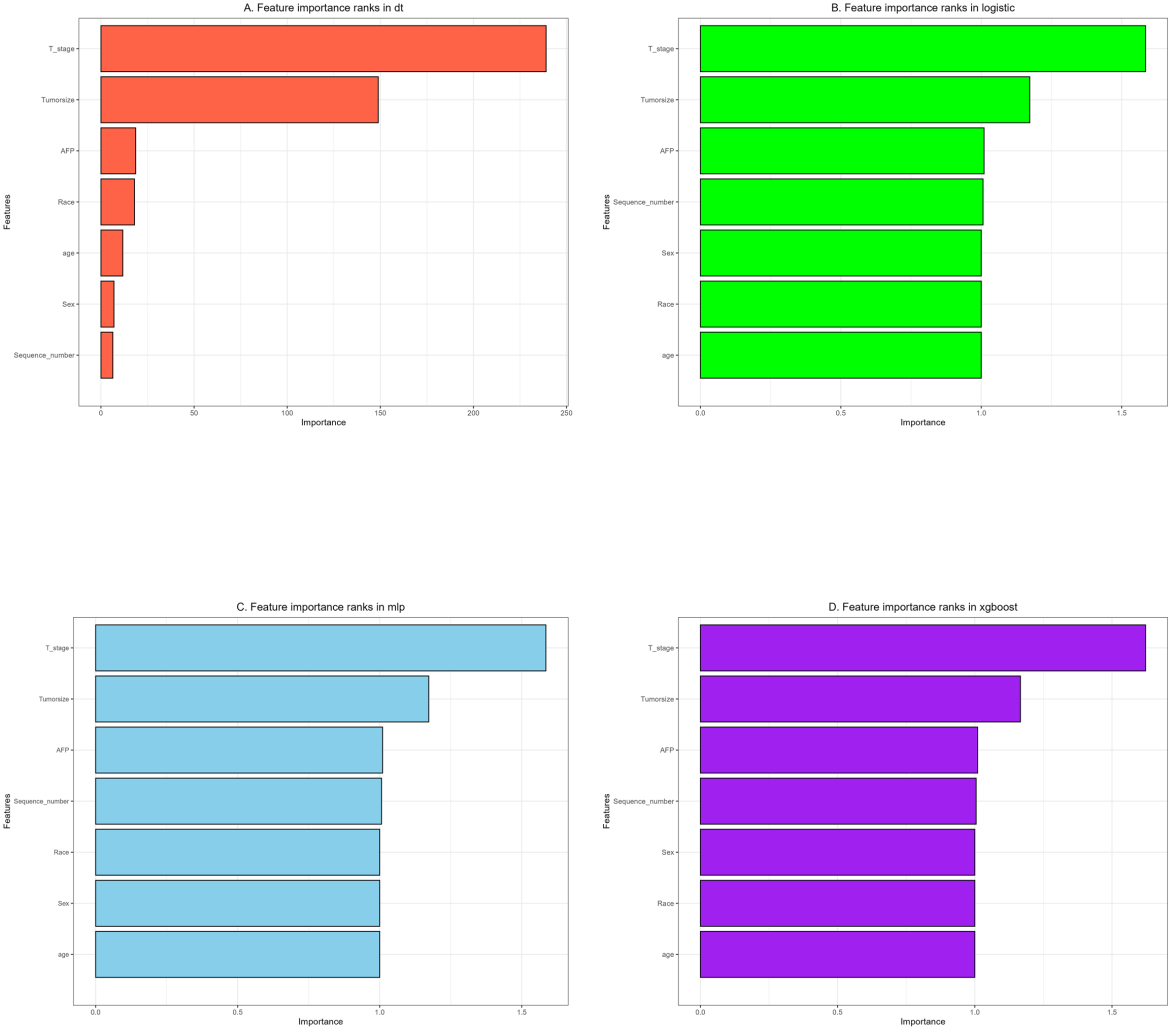
The importance of variables in each prediction model: **(A)** Decision tree (DT). **(B)** Logistic regression (LR). **(C)** Multilayer Perceptron (MLP). **(D)** Extreme gradient boosting (XGBoost).

**Figure 7:**
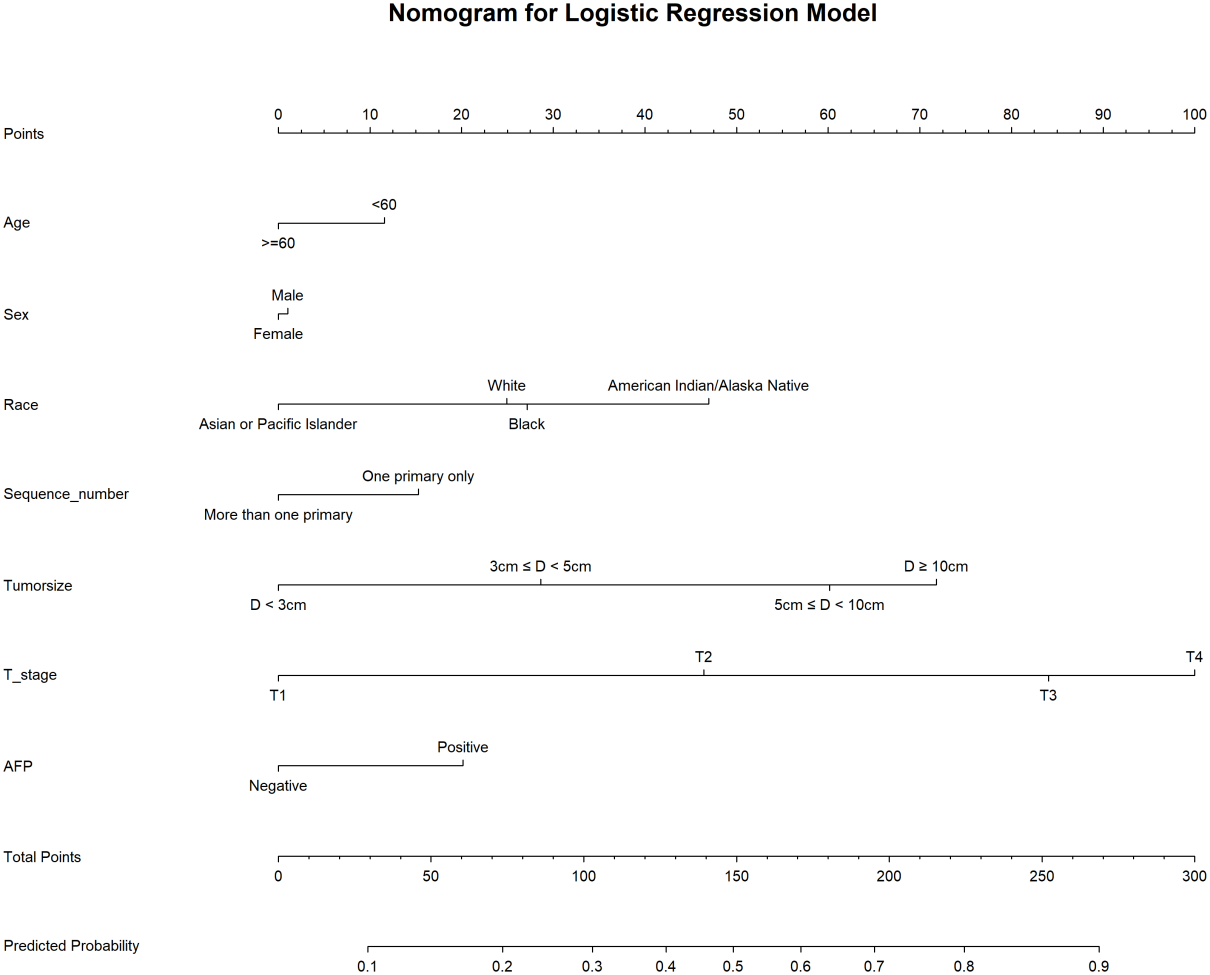
Nomogram for logistic regression model.

We used 10-fold cross-validation to optimize hyperparameters for four ML models (**Figure 3**). Comprehensive evaluation (**Table 4**) showed LR achieved the highest AUC (0.751) in the internal test set, closely followed by MLP (AUC = 0.75). ROC curves for all models on internal and external test sets are presented in **Figure 4**. Given its superior performance in the internal test set, LR was selected as the final predictive model. Its ROC curves across training, internal, and external test sets are shown in **Figure 5**. The variable importance plots for all four models (**Figure 6**) identified T stage and tumor size as the top predictors of LNM. Additionally, Given that the performance differences between the logistic regression (LR) and multilayer perceptron (MLP) models on the internal validation set were minimal, we conducted a DeLong test to compare the two models. The results indicated no significant differences in the area under the curve (AUC) between LR and MLP (AUC difference = 0.0002, p = 0.9442). Considering LR was deemed more aligned with the study’s objectives due to its interpretability advantages and comparable real-world performance, as evidenced by its consistent accuracy, sensitivity, and specificity across internal and external validation cohorts. Furthermore, a nomogram was developed based on the LR model to facilitate clinical risk assessment (**Figure 7**).

**Table 4:** Predictive performance of the algorithms’ internal and external tests.

Models		DT	Logistic	MLP	XGBoost
Training	AUC	0.766 (0.754 - 0.778)	0.767 (0.748 - 0.786)	0.769 (0.756 - 0.781)	0.754 (0.742 - 0.767)
	Accuracy	0.678 (0.671 - 0.685)	0.72 (0.712 - 0.726)	0.681 (0.674 - 0.688)	0.718 (0.711 - 0.724)
	Sensitivity	0.672 (0.664 - 0.679)	0.722 (0.715 - 0.729)	0.676 (0.668 - 0.683)	0.721 (0.713 - 0.728)
	Specificity	0.76 (0.737 - 0.781)	0.689 (0.663 - 0.716)	0.743 (0.72 - 0.769)	0.686 (0.659 - 0.712)
Internal test	AUC	0.741 (0.719 - 0.762)	0.751 (0.73 - 0.771)	0.750 (0.73 - 0.771)	0.732 (0.711 - 0.753)
	Accuracy	0.663 (0.651 - 0.673)	0.707 (0.697 - 0.717)	0.669 (0.657 - 0.679)	0.709 (0.698 - 0.719)
	Sensitivity	0.66 (0.648 - 0.672)	0.711 (0.699 - 0.721)	0.665 (0.655 - 0.677)	0.713 (0.702 - 0.724)
	Specificity	0.704 (0.662 - 0.745)	0.661 (0.622 - 0.708)	0.721 (0.676 - 0.757)	0.659 (0.614 - 0.7)
External test	AUC	0.759 (0.627 - 0.891)	0.73 (0.576 - 0.884)	0.748 (0.596 - 0.901)	0.733 (0.586 - 0.88)
	Accuracy	0.719 (0.561 - 0.807)	0.737 (0.596 - 0.825)	0.737 (0.596 - 0.825)	0.737 (0.579 - 0.825)
	Sensitivity	0.78 (0.625 - 0.889)	0.829 (0.692 - 0.927)	0.829 (0.692 - 0.923)	0.829 (0.69 - 0.927)
	Specificity	0.562 (0.286 - 0.8)	0.5 (0.222 - 0.733)	0.5 (0.22 - 0.706)	0.5 (0.267 - 0.75)

AUC, area under the curve.

### Nomogram for prediction of LNM in HCC patients

**Table 4** presents the performance of the LR model. In the internal test set, the LR model achieved an AUC of 0.751 (95%CI: 0.73 - 0.771), accuracy of 0.707 (95%CI: 0.697 - 0.717), sensitivity of 0.711 (95%CI: 0.699 - 0.721), and specificity of 0.661 (95%CI: 0.622 - 0.708). In the external test set, the corresponding metrics were 0.730 for AUC (95%CI: 0.576 - 0.884), 0.737 for accuracy (95%CI: 0.596 - 0.825), 0.829 for sensitivity (95%CI: 0.692 - 0.927), and 0.5 for specificity (95%CI: 0.222 - 0.733). Overall, the model’s performance metrics in the external test set were comparable to those in the internal test set, indicating reasonable generalizability. Although the AUC decreased slightly from 0.751 (internal) to 0.730 (external), the accuracy and specificity improved (accuracy: 0.707→0.737; specificity: 0.711→0.829), suggesting enhanced ability to identify negative cases in external data. Together with previous analyses, LR maintains stable cross-dataset performance while balancing model complexity and interpretability, supporting its selection as the optimal model. Subsequently, a nomogram for predicting LNM in HCC patients was developed based on the logistic regression model. This nomogram calculates the total score from individual variable scales to predict the probability of LNM in HCC patients (**Figure 7**). An example of the nomogram application is as follows: A patient with the following characteristics was randomly selected: age <60 years, male, White race, a single primary tumor (one primary only), tumor size ≥10 cm, T4 stage, and AFP-positive status. Based on the nomogram, the patient’s total score was 244.8 points, corresponding to a predicted LNM probability of 0.846. Pathological confirmation verified the presence of LNM, demonstrating the nomogram’s accuracy.

## Discussion

LNM in HCC represents a pivotal prognostic determinant: histologically node-positive patients demonstrate survival outcomes comparable to those with locally advanced (stage IVA) disease, and LNM markedly narrows the opportunity for curative intervention (16). Conventional detection—principally contrast-enhanced CT or MRI— suffers from suboptimal sensitivity and specificity due to HCC’s inherently low nodal metastasis incidence and confounding factors such as obesity or chronic inflammation (17). Moreover, traditional TNM-based predictive models (e.g., logistic regression) assume linearity and thus fail to capture complex, nonlinear interactions among tumor biomarkers (for example, synergistic effects between AFP levels and tumor size), resulting in biased risk stratification and potential delays in treating early-stage HCC (18).

ML addresses these challenges by integrating high-dimensional clinical data and modeling nonlinear relationships to improve predictive accuracy (19). Leveraging a cohort of 23,511 patients from the SEER database, we developed an LR model for LNM prediction achieving an AUC of 0.751. Key insights include a significant Spearman correlation between tumor size and advanced T stage—reflecting the aggressive growth kinetics of metastatic HCC (20). Methodologically, undersampling was incorporated into the preprocessing pipeline, ensuring a balanced distribution of the target variable and our approach employed 10-fold cross-validation with grid search to ensure model robustness; Despite the small size of the external cohort (n = 57), the model maintained a high sensitivity of 0.829, highlighting its reliability in excluding patients without metastasis. This helps to avoid unnecessary lymphadenectomy in low-risk cases, thereby reducing surgical morbidity (21) and minimizing center-specific bias (22).

Feature importance rankings (T stage > Tumor size > AFP) correspond closely with established molecular mechanisms governing HCC metastasis and survival outcomes (23–25). The prognostic significance of tumor size likely reflects its pro-metastatic biology: larger tumors (T2–T4) demonstrate upregulated MMP2/9 expression and enhanced exosome-mediated paracrine signaling, which facilitate lymphatic dissemination (26). Likewise, Hepatocellular carcinomas (HCCs) with positive and negative alpha-fetoprotein (AFP) exhibit distinct molecular mechanisms driving lymph node metastasis (LNM): the former is characterized by the activation of the phosphoinositol-3 kinase/protein kinase B (PI3K/AKT) pathway (27) and the upregulation of immune checkpoints, while the latter is predominantly driven by metabolic reprogramming and aberrant Wnt signaling (28). Traditionally, the presence of multiple primary tumors (MPTs) has been perceived as a high-risk indicator of cancer progression. However, our multivariate analysis in this study reveals an inverse association between the existence of MPTs and the risk of LNM (OR=0.79). This phenomenon may arise from the synergistic effects of multi-dimensional biological mechanisms, such as clonal competition that restricts the growth of metastatic subclones (29), activation of the immune microenvironment that suppresses homing, and metabolic reprogramming that diminishes invasiveness (30). Notably, race emerged as an independent predictor, with non-Asian populations (including American Indians, Blacks, and Whites) exhibiting a higher risk of LNM. Potential contributing factors may include tumor biological differences across races (such as genetic backgrounds), disparities in healthcare accessibility, and inequities in cancer screening and treatment strategies. These factors warrant further investigation and may provide valuable insights for the development of personalized oncology treatment strategies in the future.

Clinically, the LR-based nomogram offers a transformative framework for precision management of hepatocellular carcinoma. Within this model, T4 stage and tumor size ≥10 cm serve as key predictors, assigned approximately 100 and 72 points, respectively. A total score exceeding 150 points correlates with a >50% probability of lymph node metastasis, warranting comprehensive preoperative assessment.To enhance clinical applicability, we established a three-tier risk stratification system: low-risk (predicted probability <0.3), intermediate-risk (0.3–0.7), and high-risk (>0.7). This framework facilitates precise prognostic evaluation and supports individualized therapeutic decision-making.

Nonetheless, this study has important limitations. First, SEER lacks key HCC-specific variables—such as HBV/HCV viral load and Child-Pugh grade—which may omit the influence of cirrhosis-related microenvironments on LNM risk (31), Future research ought to integrate data from multiple centers, such as incorporating viral serological indicators and data on radiotherapy (32), to enhance the generalizability of the model. Second, the modest size and single-center origin of the external validation cohort (n = 57) limit generalizability; Future studies should incorporate data from multiple centers—for example, by including virological serological markers and radiotherapy-related information—to construct a larger and multi-center external validation cohort. Meanwhile, we will actively explore other accessible population-based databases to further increase the sample size and heterogeneity of the external validation set, thereby enhancing the model’s applicability and robustness across diverse populations and clinical settings. Third, despite Logistic’s interpretability advantages relative to deep learning, the inherent “black-box” nature of ML models continues to challenge clinical transparency (33).

To address these gaps, future research should: (1) By extracting radiomic features—including tumor texture, shape, and edge sharpness—from imaging data such as CT and MRI, and integrating them with lymph node texture heterogeneity and circulating tumor cell (CTC) detection, we aim to construct a comprehensive multimodal predictive platform. This approach fundamentally transforms the prediction of lymph node metastasis by shifting from traditional morphological assessment to a quantitative, dynamic, and mechanism-driven intelligent diagnostic framework (34); (2) develop streamlined ML algorithms within clinical decision support systems to enable intraoperative, real-time LNM risk assessment; and (3) validate the functional roles of key predictors (e.g., AFP) using organoid and *in vivo* models to establish a rigorous “computational prediction–experimental validation” paradigm.

## Conclusion

Using four machine learning algorithms to predict LNM in HCC, increasing age (≥60 years), male sex, American Indian, Black, and White race, larger tumor size (3cm ≤ D < 5cm, 5cm ≤ D < 10cm, D ≥ 10cm), advanced T stage (T2, T3, T4), and positive AFP levels were identified as independent risk factors. The LR model demonstrated superior predictive performance. Based on this model, a nomogram for predicting LNM in HCC patients was developed, enabling clinicians to stratify LNM risk and tailor personalized treatment strategies.

## Data Availability

The original contributions presented in the study are included in the article/supplementary material. Further inquiries can be directed to the corresponding authors.
